# CRISPR/Cas9 as Tool for Functional Study of Genes Involved in Preimplantation Embryo Development

**DOI:** 10.1371/journal.pone.0120501

**Published:** 2015-03-16

**Authors:** Jeongwoo Kwon, Suk Namgoong, Nam-Hyung Kim

**Affiliations:** Department of Animal Sciences, Chungbuk National University, Naesudong-ro, Seowon-gu, Cheongju-si 362-763, Chungcheongbuk-do, Korea; Institute of Zoology, Chinese Academy of Sciences, CHINA

## Abstract

The CRISPR/Cas9 system has proven to be an efficient gene-editing tool for genome modification of cells and organisms. However, the applicability and efficiency of this system in pig embryos have not been studied in depth. Here, we aimed to remove porcine *OCT4* function as a model case using the CRISPR/Cas9 system. Injection of Cas9 and single-guide RNA (sgRNA) against *OCT4* decreased the percentages of *OCT4*-positive embryos to 37–50% of total embryos, while ~100% of control embryos exhibited clear *OCT4* immunostaining. We assessed the mutation status near the guide sequence using polymerase chain reaction (PCR) and DNA sequencing, and a portion of blastocysts (20% in exon 2 and 50% in exon 5) had insertions/deletions near protospacer-adjacent motifs (PAMs). Different target sites had frequent deletions, but different concentrations of sgRNA made no impact. *OCT4* mRNA levels dramatically decreased at the 8-cell stage, and they were barely detectable in blastocysts, while mRNA levels of other genes, including *NANOG*, and *CDX2* were not affected. In addition, the combination of two sgRNAs led to large-scale deletion (about 1.8 kb) in the same chromosome. Next, we injected an enhanced green fluorescent protein (*eGFP*) vector targeting the *OCT4* exon with Cas9 and sgRNA to create a knockin. We confirmed *eGFP* fluorescence in blastocysts in the inner cell mass, and also checked the mutation status using PCR and DNA sequencing. A significant portion of blastocysts had *eGFP* sequence insertions near PAM sites. The CRISPR/CAS9 system provides a good tool for gene functional studies by deleting target genes in the pig.

## Introduction

The introduction of mammalian genome sequences including those of humans [1], mice [2], and domestic animals, including cows [3] and pigs [4], has increased the importance and necessity of functional genomic tools to study the roles of genes in the genome. While functional genetic studies using genome targeting technologies, including knockouts and knockins, have been achieved via homologous recombination using embryonic stem cells (ES cells) [5, 6]; however, the unavailability of ES cells in other animals, especially commercially important domestic animal species like cows and pigs, hinders the advance of functional genomic studies in these animals.

To overcome these limitations of ES cell-mediated genome targeting, genome editing technologies using novel programmable DNA endonucleases, including zinc-finger nuclease (ZFN)[7] and transcription activator-like effector nuclease (TALEN)[8], have emerged recently. Both technologies rely on the DNA recognition domain derived from ZFN or transcription activator-like effector (TALE) to specifically recognize DNA elements longer than 15 bp, and they both utilize the DNA cleavage domain from the restriction enzyme FokI for DNA cleavages [9]. Both techniques have been successfully utilized to generate gene-specific knockout or knockin in various animals, including mice [10, 11], pigs [12], and cows [13].

However, several limitations in both techniques have been reported. For example, both techniques rely on the generation of a pair of DNA-recognition modules. For ZFN, generation and selection of DNA-specific ZF modules are time-consuming processes [14]. For TALEN, the modular nature of TALE eases the difficulties of design and selection of specific DNA-binding modules [15, 16], but the repetitive nature of TALE sequences demands a complicated gene synthesis step. Therefore, the time and cost required for obtaining the proper TALEN gene is a major bottleneck to utilize these technologies to generate knockout animals [17, 18]. Another huge hurdle for both techniques is the efficiency of programmed nuclease cleavage. Because of the low cleavage efficiency of both nucleases, genome modification of livestock animals must be performed via somatic cell nuclear transfer (SCNT) [12, 13], which suffers from low efficiency caused by developmental defects from partially reprogrammed embryos [19]. Recently, genome editing via direct injection of TALEN mRNA in porcine zygotes has been reported [20], but the editing frequency in the embryo is as low as 7%.

Recently, the bacterial clustered regularly interspaced short palindromic repeat (CRISPR)/CRISPR-associated (Cas) system, which is known as the bacterial adaptive immune system that confers resistance against bacteriophages, was demonstrated as an efficient gene-targeting technology with the potential for multiplexed genome editing [21–23]. In addition to mammalian cell lines, the CRISPR/Cas9 system can be used to efficiently generate knockout or knockin organisms via zygotic injections of Cas9 and single guide RNA (sgRNA) in many organisms, including the mouse [24, 25], rat[26], zebrafish [27, 28], nematode [29], frog [30], pig [31], and monkey [32], indicating the versatility and universality of the CRISPR/Cas9 system in genome editing.

In addition to generating knockout/knockin animals, CRISPR/Cas9-mediated gene knockout/knockin can be useful to functionally characterize embryogenesis-related genes[33]. Functional characterization of genes involved in early embryogenesis using loss-of-function approaches has relied on using knockdown or antisense oligonucleotide injections [34, 35] because knockout in early embryogenesis-related genes causes embryonic lethality, and therefore maintenance of the mutant animal is impossible.

Porcine parthenogenetic preimplantation embryos have been utilized as model systems for embryogenesis [36–39]. In the present study, we tested the feasibility of the CRISPR/Cas system in studying embryogenesis-related genes in porcine parthenogenetic embryos. We chose the *OCT4/POU5f1* gene, encoding an essential transcription factor for stem cell maintenance and pluripotency [40], for the knockout/knockin study using the CRISPR/Cas9 system in porcine parthenogenetic embryos.

## Materials and Methods

### Chemicals

Unless otherwise described, all of chemicals were purchased from Sigma-Aldrich (St. Louis, MO, USA).

### Generation of Cas9 mRNA and sgRNAs

A previously described plasmid (pCAG-T3-hCAS-pA)[41], which contains human codon-optimized Cas9 plasmid cloned into a T3 promoter, was obtained from Addgene (Addgene #41815) and used to generate *Cas9* mRNA. Briefly, the plasmid was linearized with digestion of SphI and transcribed using an mMessage Machine T3 Kit (Life Technologies; Foster City, CA, USA). After transcription, template DNA was removed by treatment with Turbo-DNase (Life Technologies; Foster City, CA, USA). Resulting transcripts were purified by phenol-chloroform extraction and isopropanol precipitation and stored at −80°C until used.

Guide sequences for sgRNAs corresponding to exons of *OCT4* were selected using the CRISPR Design Tool [42] (http://crisp.mit.edu) and are shown in S1 Table. Forward polymerase chain reaction (PCR) primers containing the T7 promoter, guide sequences, and portions of the Cas9 handle were hybridized with a reverse PCR primer containing the Cas9 handle and *Streptococcus pyrogenes* terminator sequences, and this was amplified by PCR using Phusion DNA polymerase (Thermo Fisher Scientific; Waltham, MA, USA) T7 promoter primer and the terminator primers are listed in S1 Table. Resulting PCR products (123 bp) were purified with gel extraction and used as templates for *in vitro* transcription using T7 Mega-shortscript kits (Life Technologies; Foster City, CA, USA) and purified with phenol/chloroform extraction, as done with the Cas9 mRNAs.

### Generation of *OCT4*-green fluorescent protein (*GFP*) knockin constructs

To generate a C-terminal fusion of *GFP* in the porcine *OCT4* locus via homology-dependent repair (HDR), an *OCT4-GFP* fusion construct was generated using Gibson Assembly techniques [43]). Briefly, a 2103-bp fragment containing exon 5 of porcine *OCT*4 (spanning 27,266,974–27,269,103 bp at chromosome 7) was amplified using PCR and cloned into the pCRII Topo vector (Life Technology; Foster City, CA, USA).*GFP* fragments were amplified by PCR with primers containing junction sequences of *OCT4* and *eGFP*, and OCT4-pCRII Topo vector was amplified by inverse-PCR using primers corresponding to the last codon of the *OCT4* coding sequence. The amplified *OCT4* vector and *eGFP* fragments were assembled using Gibson Assembly Master Mix (New England BioLabs; Beverly, MA, USA) and transformed in *Escherichia coli* DH5α. The resulting construct, which contained the C-terminal *eGFP* fusion at the end of *OCT4* exon 5, was confirmed with DNA sequencing.

### *In vitro* porcine oocyte maturation

Prepubertal porcine ovaries were obtained from a local slaughterhouse(Farm Story dodram B&F, um0sung, chungbuk, Korea). Cumulus-oocyte complexes (COCs) were obtained from follicles that were 3–6-mm in diameter using 18-gauge microneedles. Oocytes with evenly granulated cytoplasm and a compact surrounding cumulus mass were collected and washed three times with TL-HEPES-PVA medium (Tyrode’s lactate–HEPES medium supplemented with 0.01% polyvinyl alcohol). After washing, 70–80 COCs were transferred into 500 mL of IVM medium (TCM-199; Invitrogen, Carlsbad, CA) supplemented with 20 ng/mL epidermal growth factor, 1 g/mL insulin, 75 g/mL kanamycin, 0.91 mM Na pyruvate, 0.57 mM l-cysteine, and 10% (v/v) porcine follicular fluid. After 22 h of culture, the COCs were transferred into IVM medium without hormones and cultured for an additional 22 h at 38.5°C in an atmosphere containing 5% CO_2_ and 100% humidity.

### Parthenogenetic activation and *in vitro* culture (IVC)

After maturation (44 h), cumulus cells were removed by repeated pipetting in the presence of 1 mg/mL hyaluronidase for 2–3 min. Denuded oocytes were activated by an electric pulse (1.0 kV/cm for 60 ms) in activation medium (280 mM mannitol, 0.01 mM CaCl_2_, and 0.05 mM MgCl_2_), followed by 3 h of incubation in PZM3 medium containing 2 mM cytochalasin B. About 70–80 post-activation oocytes were cultured in 4-well dishes containing 500 mL of PZM3 for 168 h. Embryo culture conditions were maintained at 38.5°C in an atmosphere containing 5% CO_2_ and 100% humidity.

### Cas9/sgRNA injections in porcine parthenogenetic zygotes

After 8 h of parthenogenetic activation, zygotes were microinjected with a mixture of Cas9 mRNA (100 ng/μL) and different concentrations of sgRNA (10, 50, or 100 ng/μL) under a Nikon TE2000-U inverted microscope (Nikon Corporation; Tokyo, Japan) using a FemtoJet microinjector (Eppendorf; Hamburg, Germany). For large-scale deletion of *OCT4*, embryos were microinjected with mixtures of Cas9 mRNA (100 ng/μL) and different concentrations of two sgRNAs (10 ng/μL sgRNA targeting exon 2 and 10 ng/μL or 100 ng/μL sgRNA targeting exon 5). To generate *OCT4-eGFP* knockin embryos, embryos were microinjected with mixtures of Cas9 mRNA (100 ng/μL), sgRNAs (10 ng/μL), and different concentrations of *OCT4-eGFP* knockin construct DNA (20, 50, or 100 ng/μL). As a control, Cas9 mRNA (100 ng/μL) without sgRNA was injected. After microinjections, zygotes were cultured in PZM3 medium for 168 h at 38.5°C in an atmosphere containing 5% CO_2_ and 100% humidity.

### Preparation of genomic DNA from blastocysts and PCR amplification

To prepare genomic DNA, blastocysts were washed twice in phosphate-buffered saline (PBS)/PVA, treated with proteinase K, and incubated at 50°C for 3 h. Portions of genomic DNA containing guide sequences for sgRNAs (exon 2 or exon 5 of porcine *OCT4*) were amplified by PCR using the PCR primers listed in Table 1 and Pfu-x DNA polymerase (Solgent; Daejun, Korea). Resulting PCR products (~300 bp) were purified by gel purification, and PCR products were cloned into the pCR-II-Topo vector (Life Technologies; Foster City, CA, USA) or sequenced directly using the PCR primers used for amplification.

**Table 1:** Developmental competence and targeting efficiency of CRISPR/Cas9 mediated Porcine *OCT4* locus. Cas9 mRNA and sgRNAs were injected in combinations of different concentrations, and cleavage and blastocyst formation rates of each groups are presented.

Gene	Cas9/sgRNA ng/μl)	Cleavage/injected zygotes	Blastocyst/injected zygotes	*OCT4* targeting fficiency	
				Immunostaning	Sequencing
***OCT4* (Exon 2)**	**100/0**	**206/264(78.03%)**	**83/264(31.44%)**	**0/15(0%)**	**0/10(0%)**
	**100/10**	**333/445(74.83%)**	**138/445(31.01%)**	**3/8(37.5%)**	**2/10(20.0%)**
	**100/50**	**310/391(79.28%)**	**123/391(31.46%)**	**7/15(46.6%)**	**5/25(20.0%)**
***OCT4* (Exon 5)**	**100/0**	**91/120(75.83%)**	**43/120(35.83%)**	**0/12(0%)**	**0/13(0%)**
	**100/10**	**149/196(76.02%)**	**65/196(33.16%)**	**13/25(52.0%)**	**12/24(50.0%)**

*OCT4* targeting efficiency was measured using the presence of *OCT4* immunostaining signal in nucleus or PCR amplification and sequencing of single blastocysts. Statistical significance was tested using chi-square test.

### Real-time reverse transcription-PCR

Porcine *OCT4*, *CDX2*, or *NANOG* gene expression levels were analyzed by real-time quantitative (q)PCR using the ΔΔCT method[28]. Total RNA was extracted from 50 oocytes using a Dynabead mRNA DIRECT Kit (Life Technologies; Foster City, CA, USA). First-strand cDNA synthesis was completed using a cDNA Synthesis Kit (Takara; Kyoto, Japan) and oligo(dT) 12–18 primers. The PCR primers used to amplify *OCT4*, *CDX2*, *and NANOG* genes are listed in S1 Table. Real-time PCR was performed with SYBR Green in a final reaction volume of 20 μL (qPCR kit, Finnzymes; Vantaa, Finland). PCR conditions were as follows: 95°C for 3 min, followed by 39 cycles of 95°C for 15 s, 57°C for 15 s, 72°C for 45 s, and a final extension of 72°C for 5 min. Finally, relative gene expression levels were quantified by normalizing to the respective *GAPDH* mRNA level. Experiments were conducted in triplicate.

### Immunofluorescence staining and confocal microscopy

For immunostaining of *OCT4* or *CDX2*, blastocysts were fixed in 4% paraformaldehyde dissolved in PBS and then transferred to a membrane permeabilization solution (0.5% Triton X-100) for 1 h. After 1 h in blocking buffer (PBS containing 1% bovine serum albumin), blastocysts were incubated overnight at 4°C with anti-OCT3/4 antibody (sc-8628, Santa Cruz Biotechnology; Santa Cruz, CA, USA) diluted 1:200, or *CDX2* antibody (MU392A-UC, BioGenex Laboratories Inc.; San Ramon, CA, USA) diluted 1:200. The blastocysts were washed three times in PBS containing 0.1% Tween 20 and 0.01% Triton X-100 for 2 min, and then the samples were co-stained with Hoechst 33342 (10 mg/mL in PBS) for 15 min and washed three times in washing buffer. Samples were mounted onto glass slides and examined using a confocal laser-scanning microscope (Zeiss LSM 710 META; Jena, Germany). At least 30 blastocysts were examined per group.

### Data analysis

For each treatment, at least three replicates were perfomed. Statistical analyses were conducted using pearson’s chi-square test or an analysis of variance(ANOVA) followed by Tukey’s multiple comparisons of means by R (R Development Core Team, Vienna, Austria). Data are expressed as mean ± standard error of the mean and p < 0.05 was considered significant.

## Results

### CRISPR/Cas9-mediated targeting of the *OCT4* gene in porcine zygotes

**Fig 1:**
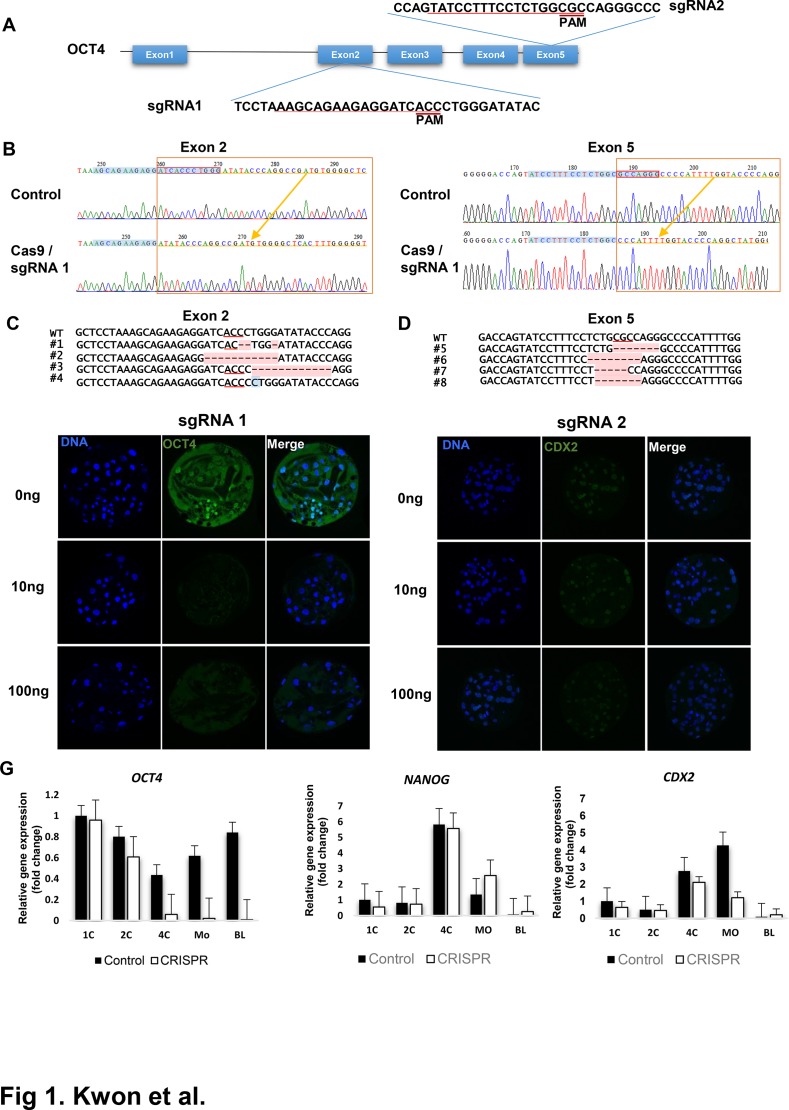
CRISPR/Cas9 mediated *OCT4* targeting in porcine embryos. (A) Design of sgRNAs for targeting exon 2 or exon 5. Guide sequences correspond to sgRNAs are marked as underlined and the protospacer adjacent motif(PAM) sites for each guide sequences are marked. (B) Sequencing of PCR amplification product confirmed the introduction of indel in exon 2 or exon 5. Locations of guide sequences are marked as blue and bases deleted in Cas9/sgRNAs injected embryos are marked in red box. (C-D) Various deletion/insertions induced by Cas9/sgRNA injections in exon 2 (C) or exon 5(D). Positions of PAM sites are marked as underlined and deleted base was marked as red. Note that insertion can be induced in some case(marked in blue). (E-F) Detections of *OCT4* and *CDX2* protein using immunostaining in targeted porcine embryos. Embryos were injected with 5–10pl of 100ng/μl of Cas9 mRNA mixed with 0, 10, 100ng/μl of sgRNA1 or sgRNA2, respectively. Location of nucleus was stained with Hoechst 33342 (blue). *OCT4*(left panel) and CDX2(right panel) are presented as green. (G) mRNA expression levels of *OCT4*, *CDX2*, and *NANOG* measured by qRT-PCR. Expression levels are presented as relative expression levels to those in control embryo at 1 cell stages. Control: Cas9 mRNA injection only; CRISPR: Cas9 mRNA and sgRNA 2 injected. In each developmental stages, 20 embryos were collected for RNA extraction.

To investigate the usefulness of the CRISPR/Cas9 system in functional characterization of genes involved in mammalian preimplantation development, we chose to use porcine parthenogenetic zygotes and *OCT4* as a model system and gene, respectively. In previous studies using mouse embryos, knockout or of *OCT4* itself did not impair early blastocyst formation [40, 44] although formation of the inner cell mass and its pluripotency were impaired [25]. To test targeting efficiency of the porcine *OCT4* gene, we chose exons 2 and 5 and selected two target regions to introduce indels via non-homologous end repair(NHEJ), as shown in Fig. 1a and Table 2. We injected 100 ng/μL Cas9 and 10, 50, or 100 ng/μL sgRNA for exon 2 and exon 5 of *OCT4* into porcine parthenogenetic zygotes, or we injected the Cas9 alone as a control. As shown in Table 1, all groups displayed similar levels of developmental competency. We checked the mutation status using pooled genomic DNA from embryos near the guide sequence by PCR and DNA sequencing. As shown in Table 1, a portion of clones (20% for exon 2 and 50% for exon 5) had an insertion/deletion near the PAM. As shown in Fig. 1b, most clones had 1–12-bp deletions near PAM regions. In some cases, single base insertions were observed (Fig. 1b). To assess *OCT4* knockout, we checked *OCT4* protein levels in the inner cell mass (ICM) of blastocysts by immunostaining with an anti-*OCT4* antibody. As shown in Table 1 and Fig. 1e, while control embryos (Cas9 injection only) had a clear *OCT4* signal in ICM regions, injection of Cas9 and sgRNAs decreased *OCT4* positivity in embryos to 37–50% of control embryos. These results indicate that injection of Cas9 and sgRNAs designed to target porcine *OCT4* successfully targeted the *OCT4* locus and depleted *OCT4* protein at 30–50% efficiency in embryos.

**Table 2:** Developmental competence and targeting efficiency of CRISPR/Cas9 mediated large-scale deletions in Porcine *OCT4* locus.

Gene	Cas9/Exon 2 sgRNA/Exon 5 sgRNA(ng/μl)	Cleavage/injected zygotes	Blastocyst/injected zygotes	Efficiency	
				Immunostaning	Sequencing
***OCT4* (Exon 2 and Exon5)**	**100/0**	**103/140(73.57%)**	**37/140(26.43%)**	**0/15(0%)**	**0/10(0%)**
	**100/10/10**	**213/280(76.07%)**	**77/280(27.50%)**	**11/23(47.82%)**	**6/50(12.0%)**

sgRNA pair corresponds to exon 2 or exon 5 were coinjected with Cas9 mRNA. Cleavage and blastocyst formation rates of each groups are presented. *OCT4* targeting efficiency was measured using the presence of *OCT4* immunostaining signal in nucleus or PCR amplification and sequencing of single blastocysts. Statistical significance was tested using chi-square test.

Next, we checked the mRNA levels of *OCT4* and other pluripotency related genes, including *NANOG*, and *CDX2* in Cas9/sgRNAs-injected and control embryos. As shown in Fig. 1g, *OCT4* mRNA levels in control embryos decreased until the 4-cell stage and increased in the 8-cell and blastocyst stages, presumably by zygotic genomic activation [45]. For embryos injected with Cas9 and sgRNA, maternal *OCT4* mRNA was sustained until the 4-cell stage, but mRNA levels dramatically decreased at the morula stage and were barely detectable in the blastocyst stage, while mRNA levels of other genes, including *NANOG*, and *CDX2* were not affected. These results suggest that *OCT4* knockout in embryos abolished *OCT4* expression in embryonic genome activation, and its effects were specific for *OCT4*.

### Large-scale genomic region deletion in the *OCT4* gene using two sgRNA pairs

**Fig 2:**
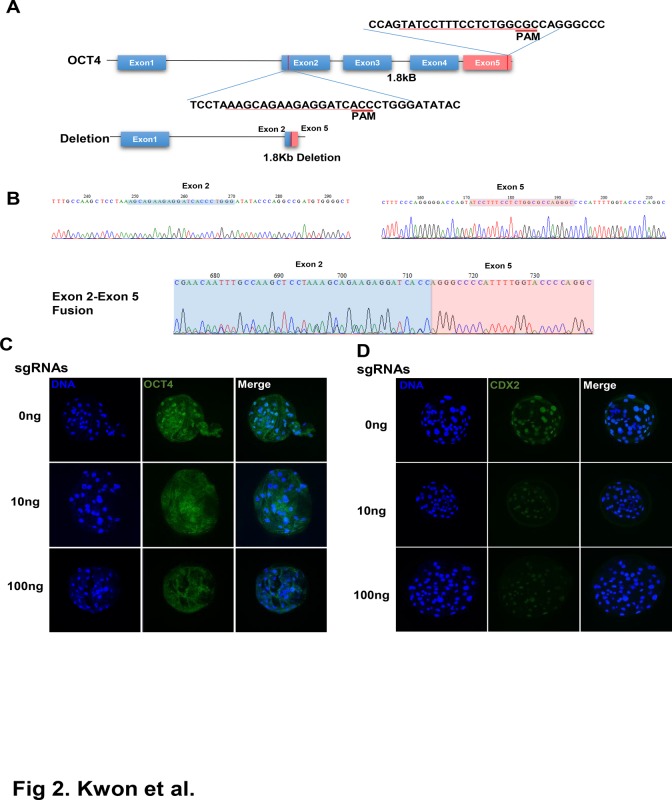
Targeted deletion of region between exon 2 and exon 5 of porcine *OCT4* locus using sgRNA pairs. (A) Location of sgRNA pair in *OCT4* locus and scheme for resulting deletion (B) Sequencing of PCR amplification product confimed the deletion of 1.8kb region between exon 2 and exon 5 of *OCT4* locus. Locations of guide sequences in exon 2 and exon 5 are marked as blue(exon 2) or red (exon 5) respectively, and resulting exon 2—exon 5 fusions are shown. (C, D) Deletion of *OCT4* and *CDX2* protein using immunostaining in targeted porcine embryos. Embryos were injected with 5–10pl of 100ng/μl of Cas9 mRNA mixed with 0, 10, 100ng/μl of sgRNA 1 and sgRNA 2, respectively. Location of nucleus was stained with Hoechst 33342 (blue). *OCT4*(C) and *CDX2* (D) are presented as green.

In addition to introduction of small indels or point mutations, CRISPR/Cas9 has been used to introduce large structural variations, including ~10-kb deletions [41] and chromosomal translocations [46, 47]. We tested the induction of large-scale deletions in the porcine genome using two sgRNAs targeting regions located in the same chromosome. As shown in Fig. 2a, the distance between the guide sequences of sgRNA 1 (targeting exon 2) and sgRNA 2 (targeting exon 5) was about 1.8 kb, and using these two sgRNA pairs, we attempted to delete 1.8-kb regions between exon 2 and exon 5 in the porcine *OCT4* gene. We injected two sgRNAs (10 ng each) with Cas9 RNA (100 ng/μL) in porcine zygotes and checked the mutation status after sgRNA/Cas9 injection. As shown in Table 2, developmental competency of embryos injected with sgRNAs and Cas9 was not significantly different from that of the control. When genomic DNA from embryos injected with both sgRNAs and Cas9 was screened for large-scale deletions, 12% (6/50) of embryos showed the expected 1.8-kb deletion, as shown in Fig. 2b. In immunostaining results shows that OCT-4 signal in blastocysts was not detected as shown in Fig. 2c. These results showed that large-scale structural variation, including exon deletions or chromosomal rearrangements, could be introduced in the porcine genome.

### Generation of *OCT4-GFP* knockins using homology-dependent repair

**Table 3:** Developmental competence and *eGFP* knockin efficiency in Porcine *OCT4* locus.

Cas9/Exon 2 sgRNA/ donor plasmid (ng/μl)	Cleavage/injected zygotes	Blastocyst/injected zygotes	Efficiency
**100/0**	**121/169 (71.60%)**	**37/140(26.43%)**	**0/8 (0%)**
**100/10/20**	**149/203(73.39%)**	**53/203(26.11%)**	**1/41(2.44%)**
**100/10/50**	**153/202(75.74%)**	**46/202(22.77%)**	**2/24(8.33%)**

Different combination of Cas9, sgRNA and donor plasmid were injected as shown. Cleavage and blastocyst formation rates of each groups are presented. *OCT4* targeting efficiency was measured by PCR amplification and sequencing of single blastocysts. Statistical significance was tested using chi-square test.

**Fig 3:**
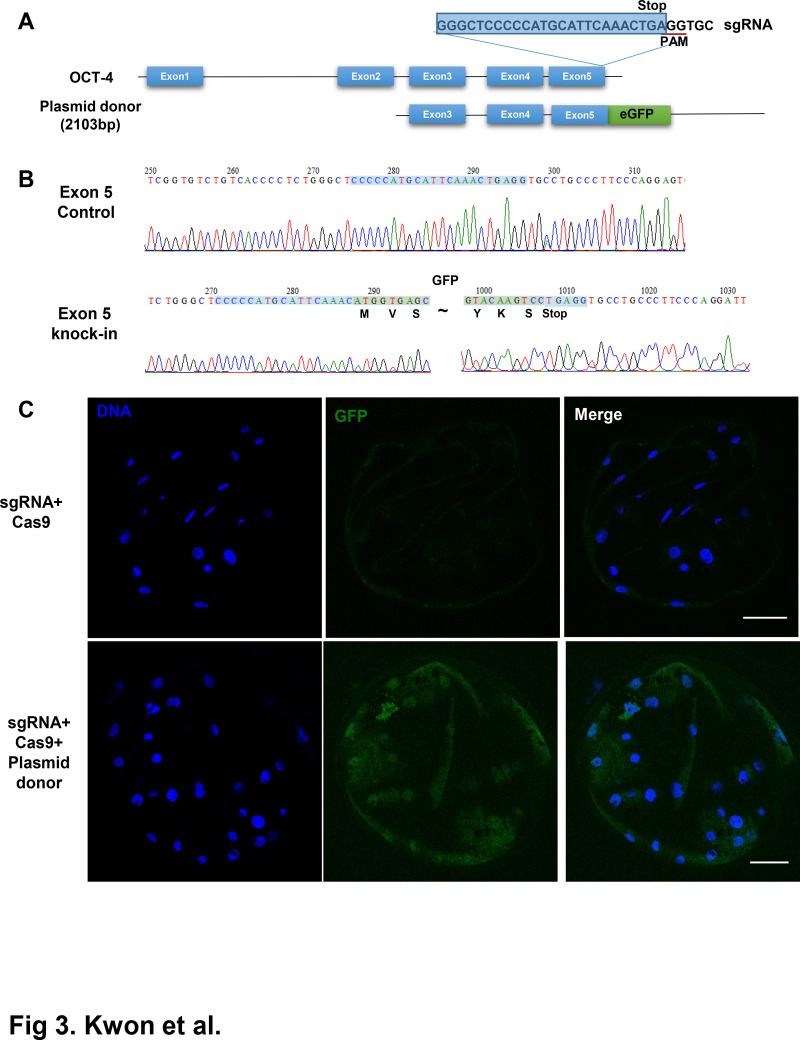
Generation of in-frame fusion of *eGFP* locus in Porcine *OCT4* using CRISPR/Cas9 mediated homology dependent repair (HDR). (A) Scheme for the HDR-mediated integration of *eGFP* into *OCT4* locus. Donor vector for HDR was consisted with the left homology arm (1kb) spanning exon 3—exon 5, *eGFP* fused after exon 5 as in-frame fusion and the right homology arm (1kb). Guide sequence for sgRNA was designed near stop codon of *OCT4* located at exon 5. Location of PAM are underlined and stop codon of *OCT4* is marked. Note that *eGFP* was inserted between stop codon and last codon of *OCT4* and PAM of sgRNA was located just after stop codon. Therefore donor vector cannot recognize and digested with Cas9/sgRNA. (B) Confirmation of insertion of *eGFP* locus in genomic DNA of porcine embryo. PCR amplification spanning exon 5 confirmed the presence of in-frame fusion of *eGFP* at the end of *OCT4* coding region. (C) Expression of *eGFP* fused *Oct4* in HDR mediated *eGFP* knockin porcine embryos. *eGFP* expression was detected byConfocal microscopy. Control (Cas9 injected) and Targeted (Cas9/sgRNA/Donor Plasmid) have been compared. Note that localization of *eGFP* signal in nucleus.

In addition to generating knockouts in the genomic locus, CRISPR/Cas9 systems have been employed to introduce precise genomic DNA knockins, including site-specific mutations, incorporation of loxP sequences, or generation of specific marker gene fusions like those with *GFP* [24, 25, 48]. We evaluated the possibility that the CRISPR/Cas9 system could be used to introduce exogenous DNA sequences in a targeted manner. We designed an *OCT4-GFP* targeting vector with a *GFP* fusion in the 3´-end of the *OCT4*-coding region, creating an in-frame fusion (Fig. 3a). Then, we injected the targeting vector as well as sgRNA/Cas9 to introduce cleavage near the 3´-end of *OCT4*. When a high concentration of targeting vector (100 ng/μL) was used, the developmental competency of injected embryos decreased (Table 3). When lower concentrations of targeting vector (20 or 50 ng/μL) were used, the developmental competencies of embryos were similar to those of the control group. We assessed the localization of *GFP* using a confocal microscope. As shown in Fig. 3c, the *GFP* signal appeared in Blastocyst-stage embryos and the *GFP* signal was detected in the ICM, indicating that the *OCT4-GFP* fusion protein was expressed in the ICM. Next, we assessed the presence of the *GFP* sequence in embryonic genomic DNA. As shown in Fig. 3b, the *eGFP* sequence was precisely integrated after the *GFP* sequence. Knockin efficiency was 2.44% (1/41) (Table 3), which is comparable with that in previous studies using mouse zygotes [25].

## Discussion

Recent advances in genome editing technologies, including ZFN, TALEN, and CRISPR/Cas9, enable precise gene targeting techniques including genetic knockout and knockin in many organisms, including domestic animals like cows [48] or pigs [49, 50].

In addition to the generation of knockout or knockin animals, CRISPR-mediated genome editing technology could be useful for functional characterization of genes involved in development [48], including pre-implantation development of mammals. However, no report has described the use of the CRISPR/Cas9 system for this purpose. To test the possibility of utilizing the CRISPR/Cas9 system in studying mammalian development, we chose porcine parthenogenetic embryos and the *OCT4* gene as models, because in vitro fertilization of porcine embryo is technically demanding[51], therefore parthenogenetic porcine embryo would be ideal model systems to test efficiency of sgRNA/Cas9 pairs before generation of knockout animal.

Our results showed that introduction of a single sgRNA/Cas9 pair yielded robust efficiency (20–50%, depending on the guide sequence) in modification. As previously reported [52, 53], cleavage and mutation efficiency were affected by the guide sequences. Specifically, Doench et al. [52] suggested that nucleotides near PAM (NGG) sites, especially at the -1 and +1 base within the NGG, were the most important elements affecting cleavage efficiency, and A/G was a favorable base for cleavage at the -1 position, and G was unfavorable at the +1 position. For sgRNA 1, which targets exon 2 of *OCT4*, the guide sequence was 5´-AGAGAAGAGGATCACCCT**GGG**CCC-3´. The NGG, GGG, is underlined. There was a T at the -1 position and an A at the +1 position. For sgRNA 2, which targets exon 5 of *OCT4*, there was an A at the -1 position and a C at the +1 position. It is noteworthy that sgRNA 2 yielded a much higher mutation frequency (50%) compared to sgRNA 1 (20%), and sgRNA 1 had the unfavorable base T at the -1 position, while sgRNA 2 had an A at that position. We do not know that these difference solely caused the difference in mutation efficiency between the two sgRNAs, but recent results concerning active sgRNA design suggest that careful choices at sgRNA sites are crucial for efficient cleavage, mutation, and achievement of high knockout efficiency, especially for functional studies of genes involved in embryogenesis.

Previous functional studies of genes involved in early embryogenesis have been performed using RNA interference or morpholino antisense oligonucleotides. A drawback of these techniques is that they ablate expression of both maternal and zygotic genes. However, as shown in Fig. 1g, knockout of *OCT4* by CRISPR/Cas9 did not affect maternal *OCT4* mRNA levels, while *OCT4* knockout only ablated embryonic gene expression. These characteristics of the CRISPR/Cas9 system would be useful to study genes involved in embryogenesis, especially those involved in zygotic genome activation.

In addition to simple knockout of a single locus, we showed that more sophisticated genome engineering, including large-scale genome deletion or knockin of *eGFP* at the *OCT4* locus to generate an *OCT4-EGFP* fusion protein, would be possible with the CRISPR/Cas9 system. Fluorescence marker proteins for visualization of biological molecules and live cell imaging are now essential tools to elucidate protein function, especially those involved in development [54, 55]. However, generation of knockin embryos with fluorescence proteins has been cumbersome and time-consuming, and use of transient fluorescent protein fusion construct expression may lead to artifacts caused from overexpression of the fluorescence protein [33]. Considering these disadvantages, fluorescent tagging of endogenous genes by CRISPR-mediated knockin and imaging of tagged endogenous protein would facilitate imaging of embryogenesis-related genes during embryogenesis. But HDR-mediated integration efficiency of *GFP* locus in *OCT4* gene is far lower than that of NHEJ-mediated knockout, as previously reported in mouse[25]. Enhancement of HDR-mediated knockin efficiency in mammalian would be essential for the generation of more sophisticated transgenesis. Recently reported techniques including NHEJ-mediated transgene integeration[56, 57] in zebrafish may be applicable in mammalian system.

In summary, we established a knockout and knockin system in porcine parthenogenetic embryos using the CRISPR/Cas9 system, and demonstrated that this system could facilitate investigation of genes involved in pre-implantation development or generation of knockin or knockout pigs.

## Supporting Information

S1 Table Primer sequences for sgRNAs, PCR sequencing, and qRT-PCR. sgRNAs: *OCT4* exon 2 and exon 5 targeting sgRNA primer; PCR sequencing: Target site mutation check primer; qRT-PCR: mRNA expression check primer.
(PDF)
