# Supplementary material for: CRISPR/Cas9 as Tool for Functional Study of Genes Involved in Preimplantation Embryo Development
Source: PLoS One. 2015 Mar 16;10(3):e0120501. doi: 10.1371/journal.pone.0120501 (PMC4361403; doi:10.1371/journal.pone.0120501)
Supplement: S1 Table — sgRNAs: OCT4 exon 2 and exon 5 targeting sgRNA primer; PCR sequencing: Target site mutation check primer; qRT-PCR: mRNA expression check primer. (PDF) [file pone.0120501.s001.pdf]

Supplementary Table 1. Primer sequences

	Primer name	Direction	Sequence (5'– 3')
sgRNAs	***OCT4 Exon2 for Knock-out***	F	TTAATACGACTCACTATAGGAGCAGAAGAGGATCACCCTGTTTTAGAGCTAGAAATAGC
	OCT4 Exon5 for Knock-out	F	GAAATTAATACGACTCACTATAGGATCCTTTCCTCTGGCGCCAGTTTTAGAGCTAGAAATAGC
	OCT4 Exon5 for Knock-in	F	GAAATTAATACGACTCACTATAGGCTCCCCCATGCATTCAAACTGGTTTTAGAGCTAGAAATAGC
	T7 long	R	AAAAGCACCGACTCGGTGCCACTTTTTCAAGTTGATAACGGACTAGCCTTATTTTAACTTGCTATTTCTAGCTCTAAAAC
	T7 short	F	GAATTAATACGACTCACTA
	T7 short	R	AAAAGCACCGACTCGGTGCC
PCR sequencing	***OCT4 Exon2 Check-1 for knock-out***	F	ACAGATACCCCCACAGACCA
	OCT4 Exon2 Check-1 for knock-out	R	CACATTTGGGGATGTTCCTT
	OCT4 Exon2 Check-2 for large sclale	F	GCAGTCCCAGGACATCAAAG
	OCT4 Exon5 Check-1 for knock-out	F	CCCCTCCCAGAGCTTATGAT
	OCT4 Exon5 Check-1 for knock-out	R	AGCCCAGAGGGGTGACAG
	OCT4 Exon5 Check-2 for large sclale	R	CTTAATCCCAAAGCCCTGGT
	eGFP for knock-in	F	CGACAACCACTACCTGAGCA

	OCT4 Exon5 Check for knock-in	R	CCCAAGAAATGGCAAAAAGA
qRT-PCR	***Nanog***	DQ447201	F:TTCCTTCCTCCATGGATCTG<br>R:ATCTGCTGGAGGCTGAGGTA
	CDX2	NM_009942	F: GATGAGGAGCAGGCTACTGG<br>R: CAGCCAAACCAGATGATA
	OCT4	NM_013633	F: CGTGGAGACTTTGCAGCCTGA<br>R: GGCGATGTAAGTGATCTGCTG
	GAPDH	AF017079	F: GGGCATGAACCATGAGAAGT<br>R: AAGCAGGGATGATGTTCTGG

(F: forward. R: reverse)
